# DIY ecology class: Transitioning field activities to an online format

**DOI:** 10.1002/ece3.6656

**Published:** 2020-08-14

**Authors:** Catherine Creech, Walter Shriner

**Affiliations:** ^1^ Mt. Hood Community College Gresham Oregon USA

**Keywords:** biodiversity sampling, COVID‐19, ecology, online teaching, quadrat, remote instruction

## Abstract

The COVID‐19 pandemic has forced the transition of many traditional face‐to‐face classes into an online format with little time to prepare best practice guidelines. In this article, we share a case study of how we adapted a group field activity into an individual laboratory assignment that can be completed during shelter‐in‐place restrictions. We conclude with ideas for future applications while staying mindful of the ways in which this pandemic has highlighted the inequities of the classroom, especially at community colleges.

## Graphical abstract

In this article, we share ways to adapt a group field activity into an individual laboratory assignment that can be completed during shelter‐in‐place restrictions. We address the tactics, difficulties, successes, and ideas for future applications while staying mindful of the ways in which this pandemic has highlighted the inequities of the classroom.

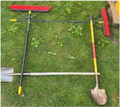

## 1 BACKGROUND

### 1.1 Teaching ecology

Ecology class is a faculty favorite for several reasons. For those of us with backgrounds in conservation biology, biodiversity, and evolution, teaching ecology is like spending time with a dear friend. We get to revisit the ideas and concepts that made us passionate about biology in the first place and our excitement is vivid, tangible, and uncontainable. Our love for the subject bubbles over when we lecture, when we explain a laboratory procedure, or when we get behind the wheel of our 15‐passenger vans full of eager students. Ecology class requires us to take ourselves a little less seriously. We kneel in the dirt, sweat while climbing the trail, and get mud under our fingernails while showing our students the interconnected beauty of the world. Ecology class is a great equalizer. Each person in the room, student and faculty alike, breathes the same air and is exposed to the same environmental factors, reminding us that we are all in this together. Our differences are less notable when we are crouching by the same stream or posing for a selfie together in front of a waterfall. Ecology class welcomes innovation and ambiguity. Students create novel experiments knowing that they are in control of the research and interpretation. The benefits of field‐based ecology experiences have been presented elsewhere (Beck & Blumer, 2012; Beltran et al., 2020; D'Amato & Krasny, 2011; Easton & Gilburn, 2012; Patrick et al., 2020); for us, ecology class highlights two major principles in the sciences: Change is the only constant, and no organism exists in isolation.

### 1.2 Challenges to moving online

Moving our ecology materials online left us with a sense of loss. We mourn the canceled fieldwork, class trips, and group hikes. We lament the loss of hands‐on learning that ecology class encourages, the walks around campus, the exploration of what is hidden in plain sight. Specifically, we rue the way remote learning highlights the inequity in our classrooms. Inequities are experienced everywhere (Cullinane, 2020; Morgan, 2020; Robinson et al., 2020), but especially in our community college setting. Students who lack computers are relegated to working from smart phones or ancient tablets. Those who lack Internet spend hours sitting in front of McDonalds in order to use the free WIFI that filters into the parking lot. Many of our students are parents, now suddenly teachers themselves trying to figure out how to navigate childcare while still submitting assignments on time. Some are newly unemployed, fighting for income, however, they can, working night shifts and overtime, or several jobs at once. Too many of our students are dealing with several of the above simultaneously. We miss the classroom chatter, the smiles, the eye contact, the ability to check in with each person, and the way we used to be able to say “I'm here for you” without having to say anything. While we fight the unmistakable unfairness of this situation as best we can, we take comfort in knowing that those two core principles of ecology are still true: Change is constant and no organism exists in isolation, even when sheltering in place forces us to be apart.

### 1.3 Current task

Critical teaching challenges during the transition to remote instruction were to bring connections and interactions to the forefront, make the material flexible and accessible, and highlight the joy of fieldwork while knowing that experiencing the outdoors during a pandemic will look different for each student. Others have documented successful efforts to incorporate field‐based ecology lessons that could be applied to a remote setting (e.g., Gastreich, 2020; Wu et al., 2016), albeit not necessarily as a result of an emergency such as the current COVID crisis. Below is an example of how we are attempting to keep the joy of discovery and connections of ecology class alive while moving our instruction online.

## 2 DIY ECOLOGY LABORATORY: AN INTRODUCTION TO BIODIVERSITY SAMPLING

### 2.1 The face‐to‐face laboratory

In our face‐to‐face classes, this laboratory is a bit of a field trip, group work exercise, and exploration trip all rolled into one. The laboratory objectives are to introduce students to the types of data collection used in biodiversity studies; give the students an opportunity to apply the scientific method to biological questions by designing experiments and using the resulting data to form and communicate a conclusion; and allow students to describe patterns of biological diversity and discuss the biotic and physical processes that lead to these patterns. To meet these objectives, we task students to answer the question “How does plant diversity change when moving from the edge of a particular habitat to the center?” The materials required are a 1 m^2^ PVC square, a reel measuring tape, and a digital camera (or a cell phone camera) for each group of four students. The data collection occurs in several habitats within a mile radius of campus, and collectively, the students are taught how to perform a transect quadrat sample before breaking off into groups. Each group finds an area to sample, collects data, photographs the plant species encountered, and then comes back to the laboratory to crunch the numbers and work toward a conclusion collectively. Back in the classroom, student groups comingle, each helping another with the math involved or how to identify a certain plant species. We come together toward the end of the period to discuss the implications of the work and extrapolate what the results mean for fellow students and the larger community. By the time class is dismissed, the students have been outside, had a hands‐on experience, worked in groups and individually, identified plants to the genus or species level, calculated species density and percent frequency, recognized trends in their data, expressed a conclusion based on the data, and connected the class material to the greater community.

### 2.2 Challenges of moving this laboratory online

There are a few aspects of this laboratory that were challenging to transfer to an online format. First was the inability to physically show students how to use a quadrat or a transect for sampling. Usually we take our students into the field, huddle around our PVC quadrats, and do the first few samples as a group, but current social distancing guidelines makes this impossible. Instead, we improvised with detailed written instructions and a YouTube video visually demonstrating the technique.

The second hurdle was how to get the physical materials to the students so they could perform the data collection. Our campus only has a few quadrats, but if students work in groups, our small set of materials is more than enough. However, with in‐person group work forbidden, our small set of materials is not adequate to supply every student with a quadrat each. Our solution to this problem was to make material procurement a do it yourself (DIY) task. We asked students to get creative and to make their own quadrats with material they could find at home or in the field. Some students used branches, bamboo sticks, or shovels, and some thought outside of the box. We saw quadrats made of pool noodles, jump ropes, leggings, floorboards, burdock roots, flow props, and large swords.

The third, and most severe, challenge of taking this laboratory online was the loss of camaraderie. In normal times, students cluster together over their samples, crawl on their hands and knees to identify plants, take photographs of pretty flowers, and group pictures to post on the Internet. Activities like these build interpersonal connections and trust. They allow students to relax and the expectations of the classroom float away once we are all outdoors. The social and emotional connections the students make are such a valuable part of the college experience. Sitting in the grass together allows bonds to be formed between students who may not have interacted in the classroom. Social distancing makes these moments feel impossible, but with the available technology, we can create new ways for our students to interact. Our solution was to add a section to our class website discussion board called the “DIY quadrat show and tell” in which students submitted photographs of their quadrats and can comment on the photographs of others much like social media. This interaction, albeit through a computer screen, allows students to see that they are not alone. It is a visual reminder that they are part of a group, that they belong somewhere, and that 30 other people had to overcome the same challenges as they did this week. These interactions remind us that our class is a community and that even if we must be physically apart we are still in this together.

### 2.3 Summary of the current online laboratory

Before collecting data, students read an overview explaining how ecological communities function, the importance of biodiversity, and the terms associated with quadrat sampling and transect sampling. A reminder to follow current social distance guidelines is bolded with the caveat that if going outside is not available to students this week an alternative assignment is available. We summarize the laboratory here and provide the complete laboratory as Appendix S1.

### 2.4 Research question

How does plant diversity change when moving from the edge of a particular habitat to the center?

### 2.5 Summary of field directions

1. Create a DIY quadrat
2. Quadrat sampling in habitat of their choosing starting at the edge of the habitat and moving toward the interior
3. Collect data and identify plants to at least the genus level
4. Create a data table for each quadrat sample that includes the species name, species density, and percentage frequency
5. Conduct a minimum of 5 quadrat samples per student and record each sample with a photograph
6. Answer the concluding questions
7. Insert the data tables and quadrat photographs into the assignment write up

### 2.6 Summary of discussion board

Students were given the following discussion board directions: “For this week's lab, you designed a home‐made quadrat, and for your discussion post, I want you to show off your creation! Please post a photo and description caption of your DIY quadrat for all your lab buddies to appreciate. Then, reply to two of your classmate's posts who had your favorite creations. I know you can't wait to see how your classmates solved this problem!”

## 3 DID IT WORK?

### 3.1 Meeting laboratory objectives

**FIGURE 1:**
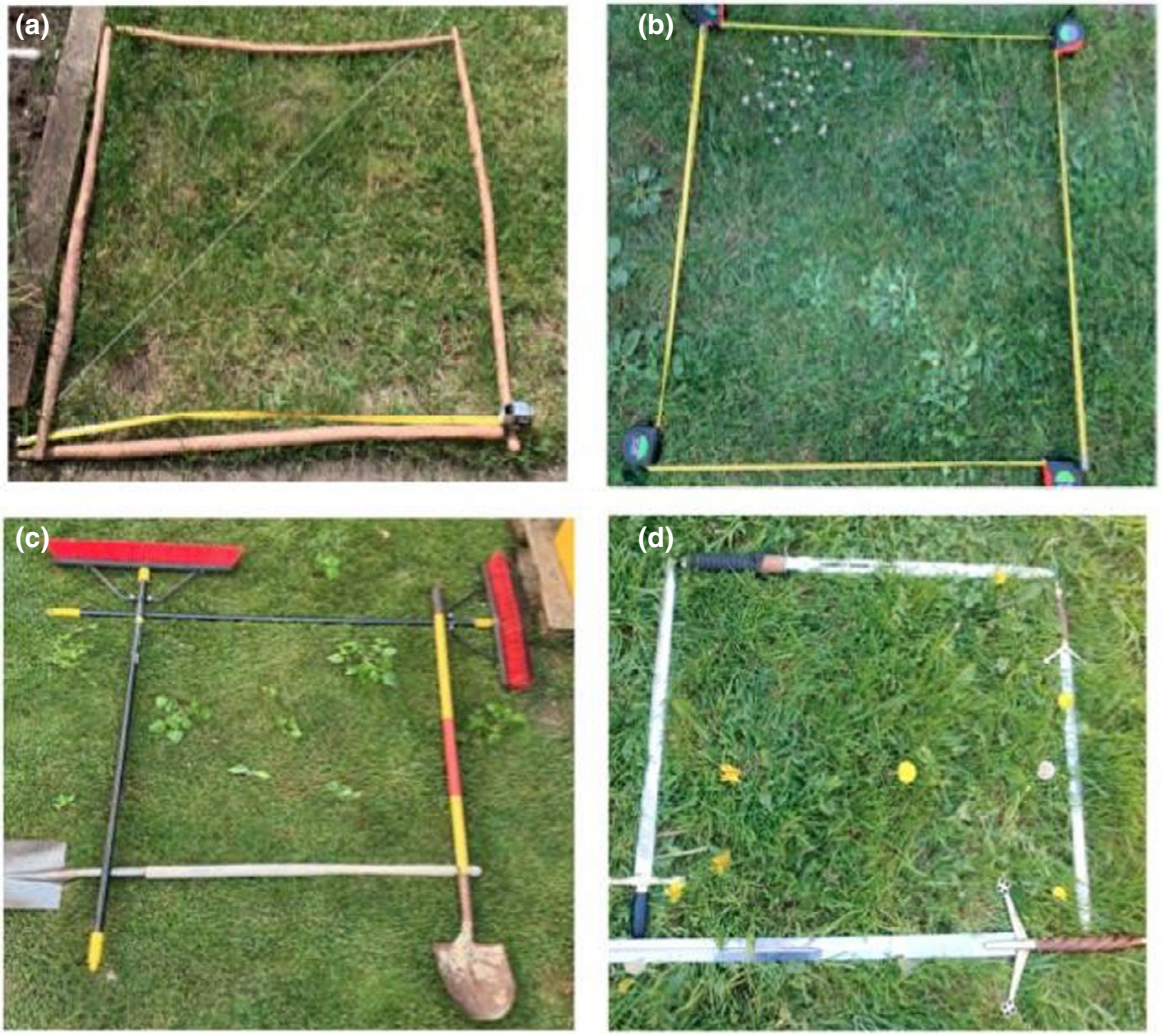
Examples of the student made quadrats composed of (a) burdock roots, (b) measuring tapes, (c) brooms and shovels, and (d) large swords

The students rose to the challenge of this online laboratory with enthusiasm and creativity (Figure 1). The data collected and results reached were of equal quality when compared with previous face‐to‐face classes. It appears that the students were able to meet the laboratory objectives and that the crux of the activity was not lost by transitioning the material to an online format.

### 3.2 Discussion board posts

Students in two joint sections of the remote ecology course (*n* = 31) generated 103 discussion post comments. Every student enrolled in the course participated in both the laboratory and the discussion post assignment. Based on the tone and word choice, it is clear that the discussion posted was used as a way to support and encourage each other, and to make social connections despite current social distancing. For example, students used the comments to praise each other's experimental design: “Creative! Being able to use what you already have is great!” “I loved that the area you chose to do your lab at was different from the typical grassy areas.” “I think it was very clever and resourceful of you to use pencils to stick into the ground to make sure the cords couldn't move!” “I think that your design looks wonderful.” “Your design looks very creative and professional.” Students also used the comments to provide each other with emotional and uplifting encouragement: “That was such a good idea!” “I am impressed, and I am also impressed at your creativity. Nice!” “WOW! This is so cool and creative!” “Wow you definitely went and created a cool square!” “I like how creative you got with using sticks, so rustic!”

Based on the responses, it seems that the students were happy overall with the online experience: “I wasn't even aware of quadrat sampling until this week. It was interesting to watch different YouTube videos and to see random people's perspectives on their DIY Quadrat.” “I thought it was interesting since I looked closely for plants I would have just ignored.” “It's amazing what we can think of if we are put in the position that we have to.”

## 4 CONCLUDING REMARKS

In the future, there are a few things we would change to improve this laboratory. First, the guidelines for the discussion posts could be more detailed and constructive. The free‐form responses were delightfully emotional and light‐hearted, but the assignment could be improved by adding clearer expectations such as follow‐up questions to reinforce the laboratory objectives or samples of successful quadrats. Second, the conclusion of the laboratory could be reworked to allow more sharing of individual results. Possibly, students could be asked to create a short PowerPoint or video explaining their answer to the research question and how they arrived at that conclusion. Third, the habitats the students sampled were not categorized. In the next version, there will be a laboratory section about identifying the type of habitat and researching what plant species are common to those areas.

Our advice to other instructors is twofold. One, be prepared for students in remote classes to be unsure of their work and need extra assurance that they are on the right track. Without being able to look over someone's shoulder in the classroom, it is easy for students to feel lost and unguided. We have tried to mitigate this with frequent emails and class zoom meetings, but there still seems to be a high level of anxiety among the students that they will accidently do something wrong. The second piece of advice is to have an alternate assignment ready for anyone that is unable to leave home. Some of our students live in urban environments with little green space accessible to them under shelter‐in‐place requirements. For this laboratory, the alternative assignments were to research different types of biodiversity sampling techniques and write a “how to” guide for five of them, or to write a literature review of two current peer‐reviewed research articles based on biodiversity. The two alternative assignments clearly have different outcomes than the field activity, but both address the topic of biodiversity and sampling techniques. Neither alternative assignment is ideal, but we need to be flexible with student limitations given the current circumstances.

In summary, the theme of change as constant is ever present in our work as instructors. We see differences between class sections, between terms, and between years. We watch our students change over the course of the class, growing more confident and developing skills. We see ourselves change and grow as well, in our work and in our expectations for ourselves. When change is as startling and stark as is it has been lately it can be easy to feel alone, but we ecologists know a secret. We know that nothing in nature exists in isolation, especially not us, and especially not now. The COVID‐19 pandemic has highlighted the ways that humans rely on each other. As we watch the world around us adjust to standing six feet apart, we know that human connection is immeasurable. We can still facilitate relationships through our instruction and foster community though digital interactions. And even though ecology class is different this term, it is still a faculty favorite.

## CONFLICT OF INTERESTS

None declared.

## AUTHOR CONTRIBUTION

**Catherine Creech:** Writing‐original draft (lead); Writing‐review & editing (lead). **Walter Shriner:** Writing‐original draft (supporting); Writing‐review & editing (supporting).

## Supporting information

Appendix S1

## Data Availability

The authors confirm that the data supporting the findings of this study are available within the article.
