# Supplementary material for: DIY ecology class: Transitioning field activities to an online format
Source: Ecol Evol. 2020 Aug 14;10(22):12437–41. doi: 10.1002/ece3.6656 (PMC7679535; doi:10.1002/ece3.6656)
Supplement: Supplementary file 1 — Appendix S1 [file ECE3-10-12437-s001.docx]

**Appendix** **I**

# Ecology Lab: An Introduction to Biodiversity Sampling

## Overview

Ecological communities are built on the interactions between the organisms and the physical environment that surrounds them. The living organisms, or biotic factors, interact as predators, prey and competitors for resources. The physical environment, or abiotic factors, such as water, sunlight, climate, temperature, soil and oxygen are part of what determine how many living creatures can be supported in the ecosystem. Plants are important in ecological communities. Not only do plants represent a large source of primary production, they also determine the potential for wildlife to exist in an area. Disturbance of vegetation can have cascading effects through an ecosystem and changing the diversity and abundance of plants will affect the other living communities, such as animals, insects, fungi, and soil microbes (Schulz et al. 2009). In particular, the more sunlight and water that is available in an area the greater the diversity of plants and animals can live in that area. For example, picture the difference between a desert and a tropical rainforest. The desert gets little water so not very many plants can grow there, which leads to few animals living there as well. In contrast, a tropical rainforest gets plenty of both sunlight and rain and hosts a wide variety of both plants and animals.

The question of how many species exists in an environment is central to understanding why it is important to promote and preserve species diversity and catalog species abundance. Promoting a singular species could leave an ecosystem at risk of failure when environmental conditions change (AMNH 2015). Biodiversity is a measure of the number and variety of different plant and animal species that live in an ecosystem. A high biodiversity can be correlated with a more stable ecosystem because there are a wider variety of resources for foraging, nesting, and seeking shelter. Abundance describes the number of individuals per species in a community. When one species is overrepresented or underrepresented in a community it can alter the stability of the ecosystem.

When studying an ecosystem, ecologists first try to survey what populations of organisms naturally live there, then measure how many of each organism lives there. This is referred to as the population density of that species. Ecologists measure population density by counting the number of each species in a sample area called a quadrat. If they count the population size in a number of quadrats chosen at random around the ecosystem, scientists can estimate how many of each species live in the whole ecosystem. The carrying capacity is how many individuals of a species that can survive in an area given the resources (food, water, and nesting sites) available (Folgia 2008). Quadrat sampling can either be random or systematic depending on the nature of your research question. In random quadrat sampling, the habitat is broken down into a grid-like system of squares and each square is assigned a number. Then a random number generator is used to select which square you will be observing. An example of systematic quadrat sampling is transect sampling, which simply means moving along a predetermined path, taking samples at predetermined regular intervals, such as from the edge of the habitat into the center. Sampling is done at regular distances to compare the biodiversity of the edge of the habitat with the interior.

In this lab you will be performing quadrat sampling to gauge the biodiversity of a habitat. You will get to choose where this habitat is but remember that you MUST follow social distancing protocols while completing this lab. If you can, complete this lab at your local park, in your yard/courtyard, or in a green area that’s legally accessible. If you can’t get outside, email me and we’ll figure out an alternative.

Be sure to read this whole lab before you head out so you know exactly what is expected of you, and take photos of what you’re doing as you go through this lab. You’ll be asked to attach your photos to the conclusion before you submit this assignment.

## Research question

How does plant diversity change when moving from the edge of a particular habitat to the center?

## Instructions

First, watch this video showing you how to conduct a quadrat sample:

<https://www.youtube.com/watch?v=RhMOCxXcDrQ>

Next, find something around your house that you can make a 1 meter x 1 meter square with. Here’s what I found at my house, but you could also use rope, string, random branches, yardsticks, or whatever else you have lying around. Feel free to get creative!


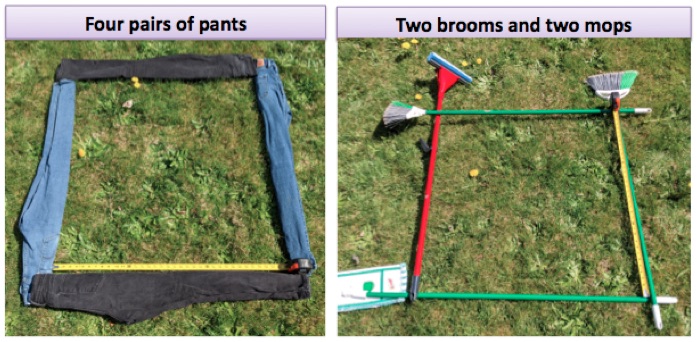


Ideally, you’ll be able to find some way to make a meter squared box, but if you can’t your quadrat can be smaller. As long as the size of your quadrat is consistent each time you use it, the experiment will work.**Be sure to take a picture of your home-made quadrat.** We’ll be using it for the discussion this week.

We’ll be using the transect method like the second technique shown in the video. This allows us to first determine the transect line that we would like to follow and use the quadrat to sample an equal amount of space at each predetermined point. That means that once you get to your habitat, you’ll choose where on the edge to start and you’ll aim toward the center of the area as you go.

Once you find a good spot, set down your quadrat to take your first data collection. To conduct the sampling within your quadrat, simply count the number of individuals of each species. You’ll need to keep track of what species are present and how many of each there are, so **you’ll need to make a data table**. Before you start, you’ll need to decide how to deal with plants that are partly in and partly out of the quadrat. One possibility is to include plants that are more than half in and exclude ones less than half in. Another is to include all the border plants on the north and east sides and exclude all of those on the south and west sides.

**Answer the following questions before moving on:**

1. What did you use to make your quadrat? Don’t forget to take a photo.
2. What rule did you adopt for plants that were partially inside the quadrat?
3. How did you decide where to conduct your first quadrat?
4. Before you collect your data, what do you think you’ll see? Will there be a higher species diversity on the edge of the habitat or the center? Will the species change as you move, or will they be consistent thought the habitat?
5. As ecologists, we need to be keen observers of the habitat we are studying. Practice paying attention by listing four biotic factors and four abiotic factors in this ecological community.

Now we’re ready to collect some data! Put down your 1m^2^ quadrat at the first point intercept. Remember to orient yourself so that your data collected are applicable to your research question. Record the types plants and number of individuals of each species in the quadrat. Yes, you do need to accurately identify your plant species to at **least to genus level**, and don’t forget to cite your sources. Common names of plants are not specific enough for this assignment. If you don’t immediately recognize the plant, you can take a picture or a sample and identify it later. Your final report should include pictures (or diagrams) of each plant you find and each quadrat you set up.

After you have completely counted all individuals and species, move 3 meters along the transect line toward the interior of the research area and repeat the procedure. Do this four times so that you have **data for five quadrat samples total**.

Determine the density of each plant species within each quadrat and create a data table showing your results.

**Density** = number of individuals of species

total individuals in quadrat

The percentage frequency is often used as a measure of abundance, especially in vegetation studies. Abundance describes the number of individuals per species in a community. Determine the percentage frequency for each species you observed and create a data table showing your results.

**Percentage frequency** = total number of quadrats containing species x 100

total number of quadrats

## Conclusion

Answer the following questions then submit your assignment to our class Blackboard page. Don’t forget to include the five questions you answered above as well!

1. What were your major results?
2. What is your answer to the research question?
3. Which plant species had the highest percentage frequency? Which had the lowest?
4. Which plant species had the highest density? Which had the lowest?
5. Is percentage frequency generally correlated with density? If no, why not? If yes, is the correlation perfect?
6. Why are changes in diversity important to determine?
7. What additional information would you like to get a more statistically accurate picture of this study population?
8. How would the diversity of this habitat be impacted if we removed a quarter of an acre of vegetation from the interior, as opposed the edge? For example, if we were to build a house in this area, would it affect the habitat more to build it on the edge or in the interior?
9. Were any of the plant species you found invasive species? Which ones? If you didn’t find an invasive species, look up a common one in your area for the next question.
10. How were the invasive species you found introduced to the area? What impact might they have on plant diversity and that of other taxa like arthropods, birds, and mammals? You are going to need to research this, so don’t forget to cite your sources.
11. Insert your data tables. If another student redid this lab assignment in the same location in a week, do you think their data tables would look the same or different? If another student redid the assignment in three months from now, would the data tables look the same? Why or why not?
12. Insert your quadrat photos. Which quadrat sample had the most plant diversity based on number of species? Which quadrat had the highest density of plant based on number of individuals?

Created by

Catherine Creech and Walter Shriner

[Catherine.creech@MHCC.edu](mailto:Catherine.creech@MHCC.edu)

[Walter.shriner@MHCC.edu](mailto:Walter.shriner@MHCC.edu)

Mt. Hood Community College

26000 S.E. Stark St.

Gresham, Oregon 97030


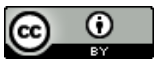
(CC BY)

References:

American Museum of Natural History (AMNH). 2015. How to Calculate Biodiversity Index. New York, New York. Available from: <http://www.amnh.org/explore/curriculum-collections/biodiversity-counts/plant-ecology/how-to-calculate-a-biodiversity-index>

Folgia, K. 2008. Study of Population Density on a Suburban Lawn. Regents Biology. Available from: <http://www.explorebiology.com/regentsbiology/labs/>

Kohn, C. 2011. Habitats & Biodiversity Lab. W. Waterford, WI. Agricultural Sciences. Available from: <http://www2.waterforduhs.k12.wi.us/staffweb/ag/Website/Kohn-For-Teachers.html>

Schulz. B., Bechtold, W., Zarnoch, S. 2009. Sampling and Estimation Procedures for the Vegetation Diversity and Structure Indicator. United States Department of Agriculture, Forest Service, Pacific Northwest Research Station, General Technical Report PNW-GTR-781. Available from: <http://www.fs.fed.us/pnw/pubs/pnw_gtr781.pdf>
